# Challenges in Diagnosing Opsoclonus-Myoclonus Syndrome in Adults

**DOI:** 10.7759/cureus.84780

**Published:** 2025-05-25

**Authors:** Janet S Lawrence

**Affiliations:** 1 Medicine, Cumberland Infirmary, Carlisle, GBR

**Keywords:** autoimmune, dizziness, oligoclonal bands, opsoclonus-myoclonus syndrome, paraneoplastic

## Abstract

Opsoclonus-myoclonus syndrome (OMS) is a rare neurological condition characterised by uncontrolled eye movements and myoclonus. The pathophysiology is autoimmune, usually due to a paraneoplastic or parainfectious cause. Studies on adult-onset OMS are not extensive, and the lack of awareness contributes to the challenge of its diagnosis. A 37-year-old female presented with nausea, dizziness, chaotic eye movements, and wide gait. Blood tests and imaging were unremarkable, but positive oligoclonal bands and elevated protein were seen in the cerebrospinal fluid analysis. She received betahistine and ondansetron as symptomatic treatment and was diagnosed with OMS. She was treated with intravenous immunoglobulin and had a slow, incomplete recovery with residual uncontrolled eye movements. A month before her presentation, she had been seen by the General Practitioner, was treated with antiemetics, and was referred to ENT. This case illustrates the importance of awareness of OMS in avoiding delay in diagnosis.

## Introduction

Opsoclonus-myoclonus syndrome (OMS) is an uncommon phenomenon in adults with autoimmune pathogenesis. Despite its rarity, it is a treatable condition with usually benign outcomes in adults [1]. Early diagnosis and treatment have been associated with a favourable prognosis [2]. Though the data collected and research on delays in OMS diagnosis are minimal, the median time between diagnosis and treatment has been reported to be 11 weeks [3]. A delay of about two months from the onset of symptoms to diagnosis has been seen to be associated with long-term neuropsychological and neurological deficits [4].

OMS is characterised by multidirectional conjugate eye movements and involuntary muscle jerking, with ataxia being a notable accompanying feature [4]. The brainstem and cerebellar theories suggest that OMS occurs due to an immune cross-reactivity involving both the humoral and cellular immune system, attacking areas such as the saccadic system that receives input from the fastigial nucleus, mesencephalic reticular formation, superior collicus, and other areas [4]. The precipitants of this immune dysfunction are thought to be paraneoplastic or infectious antigens.

Little information is known about the course of adult-onset OMS due to its low incidence. OMS is well documented in the paediatric population with an incidence of about 1 in 5,000,000 people. It is known as Kinsbourne syndrome, and it is associated with neuroblastoma and generally poor outcomes [5]. The incidence in the adult population is thought to be lower than that of the paediatric population, but no exact values have been reported in available literature. The outcomes in adults are also quite benign compared to the paediatric population.

A review of medical records of 21 adult patients (>18 years) diagnosed with OMS at the Department of Neurology, Mayo Clinic, Rochester, Minnesota, alongside a literature review of 116 OMS cases, was done [5]. Most of the patients diagnosed with OMS at the Mayo Clinic presented with nonspecific symptoms such as dizziness, nausea and vomiting, sleep disturbances, balance difficulties, mood disturbances, and tremor. All patients had opsoclonus and myoclonus, notably worsened by action. Breast cancer and lung cancer were detected in 3 of the 21 patients diagnosed with OMS at the Mayo Clinic, suggesting a paraneoplastic aetiology. In the literature review of 116 OMS cases, 60 cases were linked to cancer. Those with a paraneoplastic cause had a worse prognosis with the occurrence of neuropsychiatric symptoms than others with a supposed infectious cause.

Although there is no diagnostic marker for OMS, cerebrospinal fluid (CSF) analysis in some documented cases showed antineuronal antibodies (such as anti-ANNA-2, anti-Ri, and anti-NMDA receptors), oligoclonal bands, and elevated protein. A study by Pranzatelli et al. investigated lymphocyte subsets in the CSF and blood of children with OMS. The research found an expansion of CD19+ B cells in the CSF, with percentages reaching up to 29%. This B-cell expansion correlated with neurological severity and persisted even years after disease onset and treatment. The study suggests that CSF B-cell expansion is characteristic of OMS and may serve as a biomarker of disease activity, but this has not been standardised [6]. The mainstay of treatment for OMS is immunotherapy, and it has yielded good outcomes.

This case report provides a comprehensive overview of the patient’s clinical journey, including their presentation, diagnostic workup, treatment, and post-treatment follow-up. Although various potential causes of OMS were evaluated, the clinical picture raised suspicion of a possible infectious aetiology rather than a paraneoplastic one. The report highlights the challenges associated with the early recognition of OMS in adults and underscores the importance of heightened clinical awareness and ongoing follow-up to support timely diagnosis and effective management of this rare neurological disorder.

This case report was presented as a poster at the MANSAG 2025 Spring conference on May 03, 2025.

## Case presentation

A 37-year-old female presented to the Accident and Emergency department at Cumberland Infirmary, Carlisle, with a three-week history of nausea, dizziness, and vertigo. She had previously been prescribed chlorpromazine by her General Practitioner; however, her symptoms persisted without improvement. Over time, her condition deteriorated, leading to significant gait instability that impaired her ability to walk independently and prompted her hospital admission. There was no evidence of a flu-like syndrome; however, the patient reported a brief episode of diarrhoea before the onset of her symptoms during the systems review. She denied experiencing neck stiffness, tinnitus, loss of consciousness, or loss of hearing.

On physical examination, the patient appeared dehydrated and depressed with marked ataxia and chaotic eye movements. Neurological examination revealed intention tremors and gait ataxia. Tremors and involuntary movements were particularly noticeable in the neck and face, especially when transitioning from sitting to standing, with teeth jittering. Jerking torsional nystagmus was observed in all gaze extremes, although visual acuity appeared normal. Power, reflexes, and sensation were intact across all four limbs.

The patient’s medical history was unremarkable, with a previous obstetric history of a single, uncomplicated pregnancy, and normal vaginal delivery. Her social history indicated that she was a nonsmoker, did not consume alcohol, and lived with her family, with a WHO Performance Status of 0. There was no relevant family history of her presenting symptoms.

The primary differential diagnoses considered were vestibular neuronitis, tick-borne encephalitis, Lyme disease, autoimmune encephalitis, and functional neurological disorder.

To reach a diagnosis, several tests and imaging studies were conducted. Blood tests were largely unremarkable (Table 1). CSF analysis revealed no microbial growth or organisms. However, the presence of oligoclonal bands in the CSF IgG analysis, along with elevated protein levels (Table 2), suggested an autoimmune pathology. *Borrelia*-specific IgG and IgM antibodies, tested via enzyme-linked immunosorbent assay, were negative, effectively ruling out Lyme disease. Anti-aquaporin-2 antibodies were also undetectable, ruling out neuromyelitis optica. A comprehensive panel of anti-neuronal antibodies, including anti-Hu, anti-Yo, anti-Ri, anti-Sox1, anti-Ma2, anti-amphiphysin, anti-CV/CRMP5, anti-Zic4, anti-recoverin, and anti-Titin, was negative, further decreasing the possibility of a paraneoplastic neurological syndrome or autoimmune encephalitis.

**Table 1:** Blood test results.

	Patient’s results	Normal parameters
C-reactive protein	3	<5
Vitamin B12	443	197–771
Folate	5.1	>2.5
Hemoglobin A1c	33	20–41
Estimated glomerular filtration rate	>90	>90

**Table 2:** Cerebrospinal fluid analysis.

	Patient’s results	Normal parameters
Lactate	1.3	1.1–2.4
Glucose	2.9	2.2–4.4
Red blood cell count	<5 mm^3^	<5 mm^3^
White blood cell count	<5 mm^3^	0–5 mm^3^
Protein	0.65	0.15–0.45

**Figure 1:**
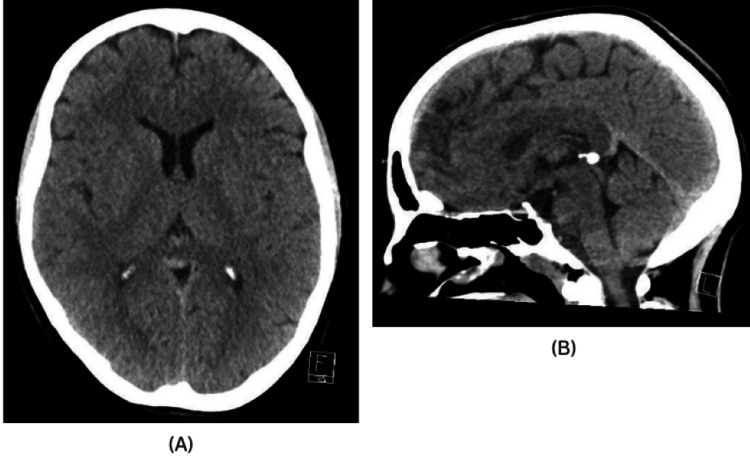
CT of the head. (A) Axial plane. (B) Sagittal plane.

Head imaging via CT showed no abnormalities (Figure 1), prompting further investigation with MRI of the brain, which similarly revealed no significant findings. A subsequent CT scan of the thorax, abdomen, and pelvis was performed, and it also showed no evidence of underlying malignancy, either above or below the diaphragm.

The diagnosis was made clinically after a thorough neurological examination by the neurologist. It was also a diagnosis of exclusion, as all other differential diagnoses were ruled out. She was treated with five days of Intravenous immunoglobulin (IVIG). She was managed with betahistine and ondansetron for vertigo and nausea, which had no effect, and underwent physiotherapy, which she reported helped when she engaged. She responded well to immunotherapy, though the response followed a slow course. It was evident six weeks later in the Neurology Clinic that she was much more stable on her feet with no tremors, but she had residual ophthalmic manifestations for which she was referred to neuro-ophthalmology. She has a much better quality of life now.

## Discussion

This young female had vestibular signs first, with one preceding episode of diarrhoea, and was treated as an ENT patient before deteriorating. The lack of awareness and the ambiguous complaints presented were the main barriers to timely diagnosis [2]. The lack of paraneoplastic features also diminished the thought of OMS in this case, unlike a clear-cut case in another case report where the patient had a teratoma and autoimmune paraneoplastic aetiology was thought of [7]; in this case, it was quite vague. OMS has been seen to present with a spectrum of vague symptoms (Table 3), with the cornerstone clinical features, opsoclonus and myoclonus, appearing later, making diagnosis challenging [8].

**Table 3:** Nonspecific symptoms in OMS. This is a non-exhaustive list containing some vague symptoms that precede OMS, obtained from the National Organization of Rare Diseases [9]. OMS = opsoclonus-myoclonus syndrome

Systemic	Gastrointestinal	Neurological	Neuropsychiatric	Vestibular
Lethargy	Nausea	Headache, gait instability	Insomnia, emotional lability, mood disturbances	Dizziness
Malaise	Vomiting	Hypotonia, tremor, sensory abnormalities	Memory loss, difficulty concentrating	Vertigo

There were some positive occurrences in this case. The appropriate tests were done promptly, and the neurologist was contacted on time with the suspicion that the symptoms were of a neurological origin. The patient was taken care of holistically; during her admission, she suffered from low mood and was seen by chaplaincy and the psychiatry liaison team. The treatment was not delayed after diagnosis was made.

Upon reflection, this syndrome can be easily overlooked, as was the case here. Meticulous history-taking and physical examination are essential to ensure timely referral to specialists. This patient had no comorbidities or specific symptoms that could narrow the diagnosis. The differential diagnoses were limited and were primarily excluded by the absence of positive findings in blood tests, CSF analysis, and imaging studies.

One of the leading differentials was autoimmune encephalitis, which was ruled out due to the absence of anti-NMDA receptor autoantibodies and negative CT scans of the thorax, abdomen, and pelvis for any malignancies. Another noteworthy differential was multiple sclerosis. Although this patient falls within the demographic commonly affected by multiple sclerosis, it was swiftly excluded based on the rapid progression of symptoms and negative MRI findings.

In idiopathic cases where no cancer or paraneoplastic association is identified, infections such as HIV, herpes simplex virus (HSV), and mumps have been reported in diagnosed OMS cases [8]. This patient tested negative for both HIV and HSV. Other potential diagnoses that could mimic OMS, such as post-infectious encephalitis, were ruled out based on the patient’s clinical state: she was conscious, alert, and oriented, with no fever or malaise and a normal CSF white blood cell count.

Given the marked ataxia observed, a diagnosis of acute cerebellar ataxia could be considered. However, this was negated by the subacute nature of the presentation, lack of imaging findings, and, most importantly, the patient’s positive response to immunotherapy.

After all the tests and scans were done, no paraneoplastic cause was found that could be linked with this case of OMS. The brief episode of diarrhoea before symptoms raises the suspicion of an infective cause.

It should also be noted that she had some CSF findings that have been documented in OMS, but due to the varying CSF analysis results in patients with OMS, the elevated protein count (Table 2) and positive oligoclonal bands found in this case were not enough to support the diagnosis of OMS. This was a clinical diagnosis made by the neurologist after ruling out other differential diagnoses.

It has been deduced that optimal neurological outcome is influenced by the timing of treatment [2]. The treatment is immunotherapy, such as rituximab, cyclophosphamide, corticosteroids, IVIG, and plasmapheresis [4] with options of monotherapy or combination therapy, although monotherapy has been seen to show a good outcome [5].

The use of intravenous immunoglobulin therapy showed promising results in this case, although the patient’s improvement was slow (over two months). It remains uncertain whether alternative or adjunctive treatments, such as cyclophosphamide, rituximab, or a prolonged course of immunoglobulin, might have offered additional benefits in reducing her residual symptoms. It is also unclear whether the delay in diagnosis contributed to the incomplete recovery or if spontaneous resolution of the opsoclonus might still occur over time. These unanswered questions highlight the importance of increased clinical awareness and the need for long-term follow-up in adult patients with OMS.

## Conclusions

OMS is a rare but treatable neurological condition. An increase in awareness of OMS could reduce the delay in diagnosis and increase the likelihood of optimal neurological outcome with timely treatment, but this is difficult due to the limited information known about this syndrome in adults and its vague presenting symptoms. At the moment, treatment options and timelines also vary, which may also influence the different outcomes seen. The data concerning the incidence of OMS in adults and the course of diagnosis are limited. There is a need for further research on OMS in adults, particularly concerning symptoms, diagnostic markers, associations with uncommon causes, the optimal treatment timeline, and the prognosis in terms of remission and residual symptoms. This would improve the ability to diagnose and counsel patients more effectively.
